# Controversy between biopsy and risk in children with proteinuria: is there a paradigm war?

**DOI:** 10.1186/s12882-024-03660-5

**Published:** 2024-07-11

**Authors:** Jingyi Yang, Xiaorong Liu

**Affiliations:** Department of Nephrology, Beijing Children’s Hospital, Capital Medical University, National Center for Children’s Health, No. 56 Nanlishi Street, Xi Cheng District, Beijing, 100045 China

**Keywords:** Chronic isolated proteinuria, Kidney biopsy, Compound heterozygous mutation

## Abstract

**Background:**

Proteinuria is a prevalent symptom of pediatric nephrology, while kidney biopsy remains the gold standard for kidney tissue analysis, and it is currently controversial. We report the rare case that the mutation in the AMN gene was considered to cause chronically isolated proteinuria and also suggest that renal biopsy should be chosen with caution in children with chronic isolated non-nephrotic levels of proteinuria and that genetic testing may be feasible for the early precise diagnosis.

**Case presentation:**

A 35-month-old boy presented with excessive urine foaming for more than half a month; his proteinuria was considered non-nephrotic range and urine protein electrophoresis was suggestive of mixed proteinuria; other than that, the investigations are non-specific. Given the child’s chronic isolated proteinuria and good renal function, we chose to refine the genetic test rather than a renal biopsy; a compound heterozygous variant was found in the AMN gene of this child which was caused by a point mutation in the father, and a partial chromosomal deletion in the mother.

**Conclusions:**

Cubilin(encoded by CUBN), amnionless(encoded by AMN), and megalin form a multiligand receptor complex; CUBN or AMN gene variants have been implicated as a hereditary cause of megaloblastic anemia, proteinuria, and neurological impairment. In the past few decades, chronic isolated proteinuria caused by CUBN gene variants is benign, non-progressive, and has normal renal function. However, the child is the first reported case of isolated proteinuria of AMN gene mutation, indicating that the earlier diagnostic genetic sequencing in an otherwise well, not nephrotic proteinuria child may be a convenient, cost-effective, and harmless option, challenging the traditional paradigm.

## Background

Proteinuria is a prevalent symptom of pediatric nephrology that frequently predicts poor renal outcomes. It is thought to be the strongest predictor of renal damage. It has traditionally been linked to progressive renal disorders such as nephrotic syndrome, glomerulonephritis, and other renal parenchymal diseases [1–3]. Kidney biopsy is a crucial diagnostic and therapeutic tool for several illnesses, including hematuria, proteinuria, acute nephritis, part of autoimmune diseases with kidney involvement, CKD of unclear etiology, and thrombotic microangiopathy. The role of renal biopsy in children with asymptomatic isolated persistent proteinuria, however, is controversial and may pose potential clinical hazards on the one hand, and financial implications for the healthcare system and the family on the other [3]. In recent years, the development of gene sequencing technologies has provided an increased alternative way to establish clinical diagnosis at reduced cost and with less harm [4]. We present the case that the mutation in the AMN gene was considered to cause impaired tubular albumin reabsorption in renal tubules, resulting in persistent isolated proteinuria. We also suggest that renal biopsy should be chosen with caution in children with chronic isolated non-nephrotic levels of proteinuria and that genetic testing may be feasible for the early precise diagnosis of childhood nephropathy and the provision of clinical guidelines for disease management as soon as possible.

## Case presentation

A 35-month-old boy presented to our outpatient clinic complaining of excessive urine foaming for more than half a month. The boy experienced no pallor or growth regression, dizziness or headache, or ataxia or falls throughout the illness. The child was born at term, weighing 4.2 kg, measuring 50 cm, and having no history of asphyxia. The mother was in good health during her pregnancy, and the child was breastfed and reached typical development milestones and motor skills development. The family had no siblings, and the parents were not consanguineous. The maternal grandfather had diabetic nephropathy, while the remaining family members had no history of kidney diseases. The boy passed hearing screening in both ears. He undertook the Childhood Autism Rating Scale (CAS) at two years old, receiving a total score of 28(The maximum score for this scale is 60; <30: non-autistic; 30–36 and less than five items with a score<3: mild to moderate autistic; >36: at least five items with a score higher than 3: severe autistic). The developmental assessment report at 25 months revealed borderline gross and fine motor skills, as well as minor language and personal-social delays, while at 28 months, the report showed borderline gross motor skills and minor language and fine motor skills delays.

The physical examination: Height 97 cm(p50), weight 13 kg(p10), head circumference 48.5 cm(p25), blood pressure 98/65mmHg. No unusual facial feature, no pallor, no edema of the face, both lower limbs and scrotum. The cardiopulmonary and abdominal examination showed no abnormality. The muscle strength of all four limbs was grade 5, muscle tone was normal, physiologic reflexes were present bilaterally, and negative pathological signs bilaterally. There was no change in urine color or volume. The external examination revealed urinary protein 2+, twenty-four-hour urinary protein quantification of 145.72 mg/24 h, urinary ultrasound revealed no abnormality, blood Hb129g/L, MCV 85.50fL, MCH 28.7pg, MCHC 336 g/L.

The child exhibited an insidious onset of disease, normal renal function(serum Creatine 19.6µmol/L, Urea nitrogen 3.88mmol/L), UACR 1.11 mg/mg, and slightly raised 24-hour urine protein, but less than 50 mg/kg, therefore proteinuria was considered to be non-nephrotic range. The serum albumin is 34.7–42 g/L, total cholesterol was normal, the ANA antibody profile, ANCA, and anti-GBM antibody were negative, there was no history of streptococcal infection, and there were no hematuria, erythema, oral ulcers, or arthralgia, so glomerulonephritic proteinuria could not be considered. His urine protein electrophoresis (quantified by SDS-AGE) was suggestive of mixed proteinuria (albumin 72.2%(153.06 mg/24 h),α1-microglobulin 6.7%(14.20 mg/24 h), β2-microglobulin 8.4%(17.80 mg/24 h)), and the Bence-Jones protein was negative, so spillage proteinuria, most commonly seen in patients with multiple myeloma, which is rare in children, is not considered. To summarize, proteinuria in this child is considered to be chronic benign persistent proteinuria with mixed components. The whole-exome gene testing was performed, and the boy regularly followed up in the nephrology clinic.

**Fig. 1:**
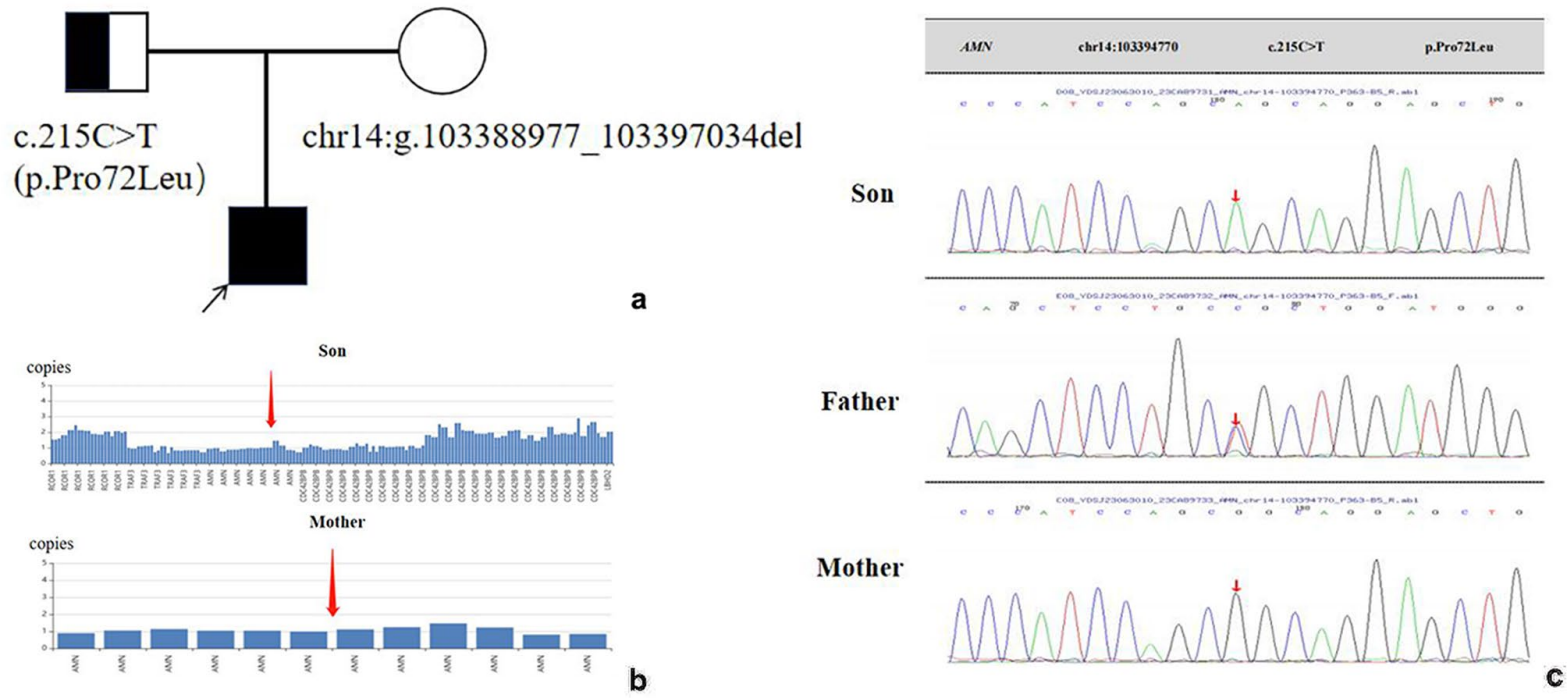
The child’s familial map and genetic mutation analysis. **a**. pedigree. **b**. Missing analysis results. **c**. point mutation analysis

This study was approved by the Medical Ethics Committee of Beijing Children’s Hospital affiliated with Capital Medical University (Approval No. [2023]-E-153-R). With the informed consent of the children and their families, 3 mL of peripheral venous blood from the children and their parents was collected separately, and the whole-exome gene test was performed at Beijing Children’s Hospital. Genetic testing (provided by MyGenotics) revealed a heterozygous deletion in the chr14:103336519–103,422,652 region and a single point mutation c.215 C > T (p.Pro72Leu) in the AMN gene, which is autosomal recessive. According to the ACMG guidelines, variant c.215 C > T (p.Pro72Leu) was initially determined to be of uncertain clinical significance, and the minor allele frequency is 0.0017; no analogous abnormalities have been documented previously. It also was predicted to be potentially harmful by REVEL, SIFT, PolyPhen_2, MutationTaster, and GERP+. The literature database contains no reports regarding the relevance of this locus. At the same time, a heterozygous deletion at 14q32.32 (chr14:103336519–103,422,652) was 86Kb at maximum and included three genes. The three genes are AMN(phenotype: Imerslund-Grasbeck syndrome 2; IGS2 OMIM: 618,882), CDC42BPB(phenotype: Chilton-Okur-Chung neurodevelopmental syndrome; CHOCNS OMIM: 619,841) and TRAF3(phenotype: encephalopathy, acute, infection-induced(herpes-specific); ILAE5 OMIM: 614,849), only AMN gene is completely deleted, while others are partial deleted, which are not reported in the ClinVar database. Family validation analysis showed that the mother of the subject had a heterozygous deletion of exon1-12 in the AMN gene. Therefore, the compound heterozygous variant in this child is caused by a point mutation in the father and a partial chromosomal deletion in the mother, which is consistent with autosomal recessive inheritance (Fig. 1). Combined with the child’s clinical manifestations and family analysis, this compound heterozygous mutation in the AMN gene may be the causative variant leading to the development of the patient, suggesting that the child may be diagnosed as chronically isolated proteinuria.

The child’s proteinuria is considered chronic and benign; there is no anemia or typical neurological impairment manifestations, and the serum vitamin B12 is normal, indicating no need for special treatment. The boy was instructed to periodically review routine urinalysis and 24-hour urine protein quantification, monitor growth indicators, and pay attention to neurological symptoms. When the boy returned to our outpatient clinic three months later for a check-up, his renal function was normal and his 24-hour urine protein quantification was essentially unchanged, ranging from 190 to 204 mg/24 hours.

## Discussion and conclusions

In the general dogma, proteinuria is always damaging. It is typically linked to unfavorable renal outcomes, and kidney biopsy is the only means to establish a definitive histological diagnosis for many of the conditions for which nephrologists care, as well as the gold standard for kidney tissue analysis [5, 6]. However, there is controversy among nephrologists worldwide, especially in pediatrics, over whether to perform renal biopsy and the appropriate timing of biopsy and ACEI application. For example, some children perform urine analysis as routine analysis or medical check-ups, the detection of proteinuria will put the pediatric nephrologists into a dilemma on whether to undertake renal biopsy. Sometimes, the normal kidney tissue or the absence of identified glomerular-related diseases will not be helpful in guiding treatment and management. However, on the contrary, it puts the children at unnecessary potential risk. For many Asian nephrologists, especially in Japan, Korea, and Taiwan, there is no doubt that children would have received a kidney biopsy [7, 8]. Starting in 1973, Japan established mass screening programs aiming at the early detection of renal disease and prompt treatment. Favorable public health outcomes have boosted consensus in these countries. While in North America, some nephrologists may wait until proteinuria is severe or a reduced glomerular filtration rate is present before considering further testing, this practice has been criticized for being detrimental to early diagnosis.

From an objective standpoint, the following are the most worrisome characteristics of kidney biopsies. First and foremost, renal biopsy is an invasive procedure, and unintended complications may occur, including bleeding, hematuria, perinephric hematoma formation, inadvertent puncture of other vessels, and arteriovenous fistula. Secondly kidney biopsy has some additional hidden costs: (1) For little children unwilling to cooperate with us, kidney biopsy frequently requires general anesthesia. Thus, the cost of this type of child will be substantially more than the average cost (5115 CNY($705.87) in our center). (2) The sampling of renal biopsy requires a particular technical and empirical judgment of the physician, and there is a certain probability of sampling failure, resulting in repeated puncture and increased invasive injury. Last but not least, the traditional methods of sedation and perpendicular access will also be traumatic for the child, even if general anesthetic and a tangential approach are available currently [9, 10].

In recent years, genetic testing, from target gene sequence, gene panel, singleton whole-exome sequencing to trio-WES, is likely to achieve confirmation of the molecular diagnosis, identifying genetic causes of proteinuria and then provide orientated prognostic information and guide management. Nevertheless, in the conventional paradigm, only genetic testing can be considered after exhaustive clinical examinations, including renal biopsy. Jayasinghe et al. have presented a cost-effectiveness analysis of targeted exome in glomerular disease [11]. She finds that non-genomic investigations(NGIs) can achieve a diagnosis in approximately 4% of children, while exome sequencing in 42%, and using WES as the first-line test can save US 1,702, highlighting the feasibility of ES as an effective tool for diagnosis and substantial cost savings [11]. It makes genetics one of the more convenient, more available, and less harmful ways to make early diagnoses [12]. In developed countries, such as Australia, kidney biopsy (without complications) costs AU$5,300 (US 3,700). In contrast, the cost of exome sequencing is AU$2,300 (US 1,600), and the standard turnaround time is three months; in the United States, exome sequencing fluctuates between US 499 and US 585, but the insurance coverage for genetic testing can limit its use in rare diseases; in Japan, the cost of whole-exon genetic testing is 10 billion yen(US 643), and the cost of renal biopsy depends more on the cost of consumables and pathology, which may be different in these countries. We also examined the gap between the cost of kidney biopsy and the cost of genetic testing in our center to provide a viewpoint from a developing country. In our facility, the average cost and time cost between kidney biopsy and genetic testing are nearly equal; nevertheless, kidney biopsy has some additional hidden costs: (1) For little children who are unwilling to cooperate with us, kidney biopsy frequently requires general anesthesia; thus the cost of this type of children will be substantially more than the average cost (5115 CNY(US 705.8)). (2) The sampling of renal biopsy requires a certain technical and empirical judgment of the physician, and there is a certain probability of sampling failure, resulting in repeated puncture and increased invasive injury.

In childhood, proteinuria can be categorized as transient or intermittent, upright, and persistent. Transient and upright proteinuria is usually benign, caused by fever, exercise, stress, and hypovolemia, and the latter is seen in children with left venous compression. Persistent proteinuria requires further evaluation for underlying etiology. Persistent asymptomatic isolated proteinuria is not common in children because children who suffer from injured podocytes or with sclerotic renal damage will experience a significant loss of urinary protein. Hama et al. have proposed that uP/Cr ≥ 0.5 g/g will be an adequate renal biopsy criterion in children with asymptomatic constant isolated proteinuria, However, it must still be validated with large sample cohort data [13].

Our child was evaluated for renal function, electrolytes, UACR, 24-hour urine protein, urine protein electrophoresis, and autoimmune antibody-related tests after the initial finding of proteinuria. The results showed that the child had persistent isolated non-nephrotic levels of proteinuria. Genetic testing provided direction for our diagnosis and treatment. The child had a paternally derived point mutation and a maternally derived heterozygous chromosomal deletion in the AMN gene, which may have led to the final consideration of a renal limit form of Imerslund-Grasbeck syndrome(IGS) type 2 in the child. As benign and non-progressive, this type of proteinuria is of normal kidney function, so we finally chose not to promptly perform further renal biopsy but follow-up for a long period of time. However, suppose the genetic result is negative, given that the child is a 2-year-old toddler. In that case, we will do regular follow-ups every 3–6 months. If the child develops diminished renal function, we will evaluate the diagnosis of the underlying cause based on renal pathology.

Amnionless, the subunit of the Cubam receptor, is encoded by the AMN gene. This gene is also known as the cause of IGS type 2. Imerslund-Grasbeck syndrome, also known as familial selective vitamin B12 malabsorption syndrome, typically presents as a triad of megaloblastic anemia, proteinuria, and neurological impairment and is caused by mutations in the Cubam receptor subunit of the multiligand receptor complex. Depending on the mutant subunit, IGS can be divided into two categories: IGS type 1 (Cubilin, encoded by the CUBN gene [Genbank NG_008967]) and IGS type 2 (Amnionless, encoded by the AMN gene [Genbank NG_008276]). We have to state that our patient can not be diagnosed as IGS type 2 with the lack of a typical triad of signs. However, the mechanism by which mutations in the AMN gene cause the amnionless variant to perform isolated proteinuria remains unidentified. The Cubam receptor can be expressed in a variety of tissues, including the ileum, kidneys, and yolk sacs [14], and is primarily localized in the terminal mucosa of the ileum, where it binds to the vitamin B12-IF receptor to promote the absorption of vitamin B12. It can also be found in the proximal renal tubule, where it promotes endocytosis of albumin through the direct interactions of cubilin and the indirect interactions of Megalin and mediates the reabsorption of low molecular proteins [15]. To the best of our knowledge, persistent proteinuria caused by CUBN or AMN gene variants is benign, non-progressive, and not accompanied by a gradual loss of renal function. A recent study found that megalin/cubilin knockout mice had blocked proximal tubular albumin uptake. However, plasma albumin levels remain unaffected, suggesting a limited effect of megalin/cubilin on albumin homeostasis, which may explain the presence of persistent proteinuria in children with IGS but normal renal function [16].

Normally, one amnionless and the N-terminal hydrophobic regions of three cubilin subunits are anchored to the membrane in the β-helix-β-helix association. On the other hand, Cubilin posttranslational modification and apical membrane expression, are highly dependent on normal amnionless function and localization. It is, therefore, reasonable to assume that mutations in CUBN or AMN gene with proteinuric phenotypes may affect the general expression or that the mutations themselves can lead to cubilin truncation or ultimately affect the clinical phenotype by affecting the cell surface expression of cubilin in patients with AMN mutations [17, 18] Human C-terminal CUBN variants are proven to associate with chronic proteinuria and normal renal function through podocyte apoptosis via the PI-3 K/PKB pathway which leads to the reduction ability of cubilin-binding with albumin and more free albumin binding with megalin [19]. Bedin et al. report 39 patients of chronic isolated proteinuria and normal function with CUBN variants, while most of them present minimal change disease or no lesions and only two are in the early stage of FSGS [16]. Yang et al. present three children with isolated proteinuria caused by CUBN gene mutation, while focal segmental glomerulosclerosis is also found in these children. Eventually, with the administration of tacrolimus, they achieved a further significant reduction in urinary protein/creatinine [20]. However, there is a lack of reports of chronic isolated proteinuria due to mutations in the AMN gene. Two issues are calling for the pediatric nephrologists’ attention for such children. First, do patients with non-nephrotic range proteinuria and normal renal function, like patients with CUBN or AMN gene variants, need a kidney biopsy, and if so, when? Secondly, do patients with CUBN or AMN gene variants need tacrolimus for treatment, and if so, when and how long? Unfortunately, there is a dearth of research on the association between the AMN or CUBN gene mutation and the clinical phenotype, management, and prognosis, necessitating the collection of further multicenter and large-sample population data and follow-up.

Currently, intramuscular or oral vitamin B12 therapy is the preferred treatment for children with IGS, and the efficacy of oral vitamin B12 has been demonstrated in recent years in studies of IGS beagles. Our child, a renal limited form of IGS type 2 who was not treated medically for vitamin B12 deficiency, requires regular follow-up in the nephrology clinic and also needs to be alerted to the possibility of new clinical phenotypes that may accompany the onset of the disease with age.

In conclusion, our case challenges the traditional paradigm, adding credence to the notion that the earlier selection of diagnostic genetic sequencing in an otherwise well, not nephrotic proteinuria child precedes invasive procedures, which could provide a more effective tool for diagnosis and substantial cost savings, help avoid the unwarranted damage caused by renal biopsy and improve outcomes. In the meantime, the case has broadened our clinical understanding of the spectrum of AMN gene mutation disorders. Meanwhile, we also have some limitations. We need to state that the overall genetic conclusion should be cautious due to VUS (variants of unknown significance), even biallelic allele mutations. Unfortunately, our child was not functionally validated to verify changes in the target gene at the DNA, RNA, and protein levels and to monitor the altered biological processes, which is critical.

## Data Availability

The data that support the findings of this study are available on request from the corresponding author. The raw sequence data reported in this paper have been deposited in the Genome Sequence Archive (Genomics, Proteomics & Bioinformatics 2021) in National Genomics Data Center (Nucleic Acids Res 2022), China National Center for Bioinformation/Beijing Institute of Genomics, Chinese Academy of Sciences (GSA-Human: HRA007728) that are publicly accessible at https://ngdc.cncb.ac.cn/gsa-human [21, 22].

## Abbreviations

CKD: Chronic kidney disease
ANA: Anti-nuclear antibody
ANCA: Anti-neutrophil cytoplasmic antibody
anti-GBM: Anti-glomerular basement membrane
UACR: Urinary albumin/creatine ratio
ACEI: Angiotensin-converting enzyme inhibitors
WES: whole exome sequencing
IGS: Imerslund-Grasbeck syndrome
